# Various Allotropes of Diamond Nanoparticles Generated in the Gas Phase during Hot Filament Chemical Vapor Deposition

**DOI:** 10.3390/nano10122504

**Published:** 2020-12-14

**Authors:** Hwan-Young Kim, Da-Seul Kim, Kun-Su Kim, Nong-Moon Hwang

**Affiliations:** 1 Department of Materials Science and Engineering, Seoul National University, 1 Gwanak-ro, Gwanak-gu, Seoul 08826, Korea; welcome777@snu.ac.kr (H.-Y.K.); daseul@snu.ac.kr (D.-S.K.); lstatsl@snu.ac.kr (K.-S.K.); 2 Research Institute of Advanced Materials, 599 Gwanak-ro, Gwanak-gu, Seoul 08826, Korea

**Keywords:** HFCVD, nanodiamonds, cubic diamond, n-diamond, hexagonal diamond, i-carbon

## Abstract

Diamond nanoparticles have been synthesized using various methods. Nanodiamonds generated in the gas phase were captured on the membrane of a transmission electron microscope grid during a hot filament chemical vapor deposition (HFCVD) diamond process. In total, ~600 nanoparticles, which were captured for 10 s in six conditions of the capture temperatures of 900 °C, 600 °C and 300 °C and the gas mixtures of 1% CH_4_-99% H_2_ and 3% CH_4_-97% H_2_, were analyzed for phase identification using high-resolution transmission electron microscopy and fast Fourier transformation. Hexagonal diamond, i-carbon, n-diamond, and cubic diamond were identified. The observation of two or more carbon allotropes captured on the same membrane suggested their coexistence in the gas phase during HFCVD. The crystal structure of carbon allotropes was related to the size of the nanodiamond. The crystal structure of the nanoparticles affected the crystal structure of diamond deposited for 8 h. Confirmation of various carbon allotropes provides new insight into the nanodiamond synthesis in the gas phase and the growth mechanism of HFCVD diamond.

## 1. Introduction

Diamond has been synthesized using high-pressure/high-temperature methods [1], and plasma-discharge-stimulated chemical vapor deposition (CVD) [2,3] and hot filament CVD (HFCVD) [4]. In addition to the synthesis of diamond bulks or films, nanodiamonds could be synthesized by detonation [5], laser ablation [6], plasma-assisted CVD [7], ion irradiation of graphite [8], electron irradiation of carbon onions [9], ball milling of high-pressure/high-temperature diamond [10] and ultrasound cavitation [11]. Detonation, laser ablation, and ball milling methods are used commercially, with detonation being the most common approach. Nanodiamonds are applied to biological studies, medical diagnostics and quantum technologies because the optical centers in diamond offer well-defined optical transitions and long-lived spin quantum states [12,13,14]. However, detonation nanodiamonds tend to be agglomerated and to include impurity defects, making it difficult for them to be applied to photoluminescence [15,16].

For applications of nanodiamonds, it is necessary to synthesize non-agglomerated high quality nanodiamonds. Park et al. [17] succeeded to confirm the generation of non-agglomerated diamond nanoparticles in the gas phase under the synthesis condition of diamond films by HFCVD. Their result showed that non-agglomerated high quality nanodiamonds can be synthesized using a HFCVD reactor. As the extension of their work, here we studied the crystal structure of nanodiamonds and how the crystal structure is affected by the processing condition. For this, we captured nanodiamonds generated in the gas phase on a graphene membrane of a transmission electron microscope (TEM) grid during HFCVD using a capturing apparatus under various processing conditions. The analysis of the nanoparticles by high resolution TEM (HR-TEM) and fast Fourier transformation (FFT) revealed four carbon allotropes: i-carbon, hexagonal diamond, n-diamond, and cubic diamond. By comparing the size distribution of the captured nanoparticles at capture temperatures of 900 °C, 600 °C and 300 °C at the gas mixtures of 1% CH_4_-99% H_2_ and 3% CH_4_-97% H_2_, we studied the relation between the size of nanodiamonds and the crystal structure. In addition, the crystal structure of the nanoparticles was shown to be related to the crystal structure of the deposited diamond.

## 2. Materials and Methods

### 2.1. Preparation of Nanoparticles

**Figure 1:**
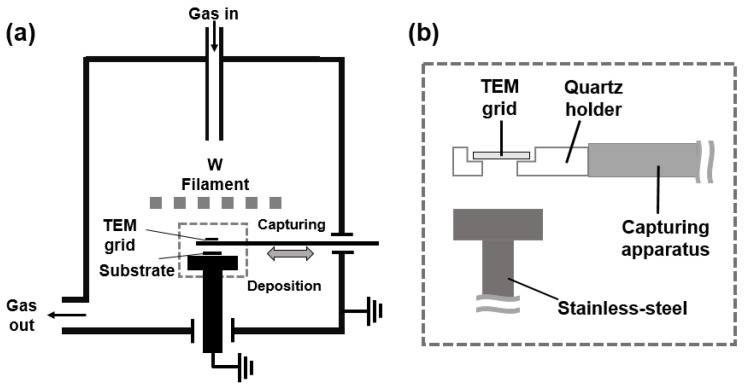
Schematics of (**a**) the hot filament chemical vapor deposition (HFCVD) reactor with the apparatus for capturing carbon nanoparticles on the graphene membrane of the TEM grid and (**b**) the capturing apparatus.

The HFCVD reactor with the capturing system is shown schematically in Figure 1. The filament consisted of three tungsten wires of 0.5 mmø, which were twisted into a nine-turn coil of 8 mmø. The filament temperature was 2100 °C and the reactor pressure was 20 Torr. CH_4_ and H_2_ were supplied as a gas mixture at 1 standard cubic centimeter per minute (sccm) and 99 sccm or at 3 sccm and 97 sccm, respectively, using a mass flow controller.

When nanoparticles were captured on the membrane of a TEM grid at high temperature such as 900 °C, capturing was difficult due to the thermal damage or etching of carbon by atomic hydrogen. Several membranes were tested. The SiO membrane (SiO Type-A, Ted Pella, Inc., Redding, CA, USA) proved very vulnerable to thermal damage above ~600 °C. The carbon membrane (ultrathin carbon type-A; Ted Pella, Inc.) was easily etched away at a capture temperature of 900 °C. After many trials, we determined that a graphene membrane was most suitable for nanoparticle capture, as it is conducting and can withstand thermal damage over the capture time of 10 s. Additionally, some of the graphene membrane remained unetched within the 10 s timeframe, which made it possible to capture diamond nanoparticles even at 900 °C.

A graphene membrane (6–8 layers of graphene film; Ted Pella, Inc.) was used to capture the diamond nanoparticles. A schematic of the capturing apparatus is shown in Figure 1. The capturing apparatus could be pushed to the capture zone as needed and retracted back toward the chamber wall. The distance between the hot filament and the capture zone was varied as 6 mm, 30 mm and 50 mm, where the temperature of the capture zone was, respectively, 900 ± 50 °C, 600 ± 50 °C and 300 ± 50 °C. Before capturing, the filament was carburized at 2100 °C for 24 h at 1% CH_4_-99% H_2_ when capturing was conducted at 1% CH_4_-99% H_2_ and at 3% CH_4_-97% H_2_ when capturing was conducted at 3% CH_4_-97% H_2_. To increase the reproducibility of the experiments, capturing was conducted after supplying the gas mixture of CH_4_ and H_2_ for 30 min at the filament temperature of 2100 °C. The quartz holder loaded with the TEM grid was pushed to the capture zone, and the grid was exposed for a capturing time of 10 s. After that, the quartz holder was pulled from the capture zone to the chamber wall. Captured diamond nanoparticles were analyzed by HR-TEM (JEM-2100F; JEOL Ltd., Tokyo, Japan). The grid was placed on the quartz holder, and the holder was connected to the capturing apparatus.

### 2.2. Analysis of Nanoparticles 

Some captured carbon nanoparticles were polycrystalline or aggregated. Most of them were single crystals, which were used to identify the phase. From the FFT image of each nanoparticle, lattice parameters and the angle between lattices were determined, based on Joint Committee on Powder Diffraction Standards (JCPDS) data of the reported carbon allotropes. In identifying the phase of single-crystalline carbon nanoparticles, we encountered two difficulties. The first was the coexistence of allotropes of carbon nanoparticles, and the second was that the different carbon allotropes have the same d-spacing value. As a result, several d-spacing values and lattice angles of the carbon nanoparticles needed to be evaluated. Out of nanoparticles captured on the membrane at each temperature of 900 °C, 600 °C and 300 °C, ~100 nanoparticles were chosen to determine the size and d-spacing by DigitalMicrograph (Gatan, Inc., Pleasanton, CA, USA). One hundred fifty d-spacings were obtained from ~100 nanoparticles, which was repeated 6 times for 6 experimental conditions of capture temperatures of 900 ± 50 °C, 600 ± 50 °C and 300 ± 50 °C and the gas mixtures of 1% CH_4_-99% H_2_ and 3% CH_4_-97% H_2_. The d-spacings were classified from JCPDS of the reported carbon allotropes. All lattice angles of the reported carbon structures, from i-carbon to cubic diamond, were used to identify the crystal structure of individual nanoparticles. 

### 2.3. Current Measurement 

Using a picoammeter (model 6487; Keithley Instruments, Cleveland, OH, USA), we measured the current between the substrate and the ground, which would represent the density of electrons emitted from the hot filament. The feedthrough, which was connected to the picoammeter, was placed 30 mm below the filament. The number density of nanoparticles captured on the graphene membrane was measured by ImageJ software (ImageJ, National Institutes of Health, Bethesda, MD, USA).

### 2.4. Deposition of Diamond Particles 

Cubic diamond and n-diamond allotropes were dominant in the gas mixture of 1% CH_4_-99% H_2_, whereas nanoparticles of i-carbon allotropes were more dominant in the gas mixture of 3% CH_4_-97% H_2_. The deposition behavior of diamond depends on which allotropic nanoparticle was dominant in the gas phase. To study this, diamond particles were deposited for 8 h on Si substrates under filament and substrate temperatures of 2100 °C and 900 °C, respectively. To observe individual diamond particles, the Si substrate was not pretreated because the pretreatment tends to produce films. The Si substrate was placed 6 mm below the hot filament. The microstructure of the deposited diamond particles was observed by field-emission scanning electron microscopy (FE-SEM; SU70; Hitachi Ltd., Tokyo, Japan).

## 3. Results and Discussion

### 3.1. Identification of Nanoparticles

**Figure 2:**
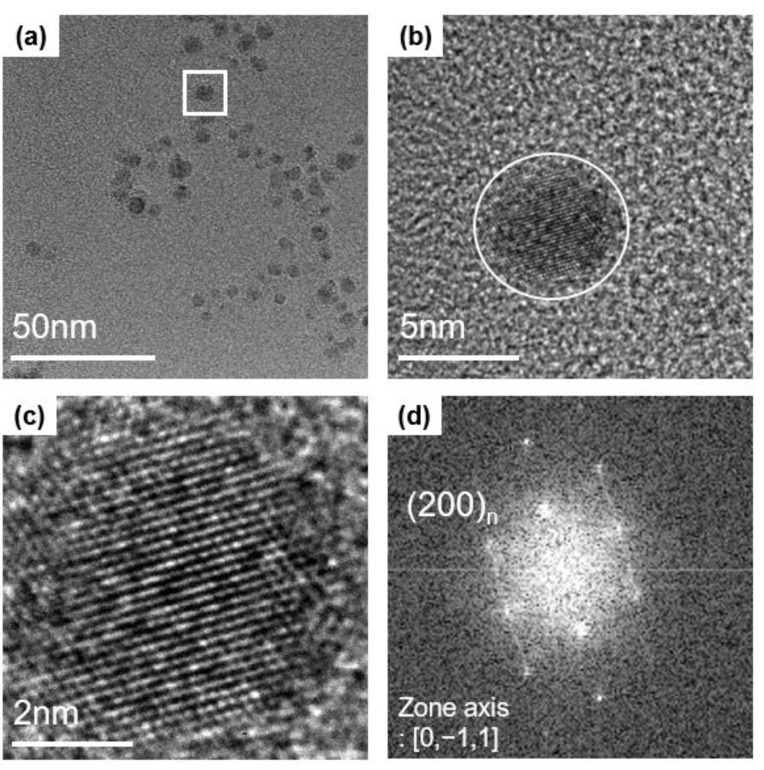
(**a**) TEM image of nanoparticles captured on the graphene membrane of the TEM Cu grid with a capture time of 10 s, (**b**) high resolution TEM (HR-TEM) image of the white square in (**a**), (**c**) a higher magnification of the nanoparticle in the white circle of (**b**,**d**) the fast Fourier transformation (FFT) image of (**c**).

After analyzing ~600 captured nanoparticles, we could identify four kinds of carbon allotropes: i-carbon, hexagonal diamond, n-diamond and cubic diamond. As an example, Figure 2a shows a TEM image of nanoparticles on the graphene membrane of the Cu grid for a capture time of 10 s and capture temperature of 900 °C, using a filament temperature of 2100 °C and a gas mixture of 1% CH_4_-99% H_2_. Figure 2a shows that nanoparticles of 2~7 nm are distributed randomly. The HR-TEM image in Figure 2b shows the magnified image of the white square in Figure 2a. Figure 2c is the magnified image of the nanoparticle in the white circle of Figure 2b, showing the lattice image of the nanoparticle with a diameter of ~4.9 nm. The FFT image recorded from the nanoparticle in Figure 2c is shown in Figure 2d. The FFT image in Figure 2d shows the (200) forbidden plane, indicating that the nanoparticle has an n-diamond structure. This is the way that observed nanoparticles were identified and classified.

**Figure 3:**
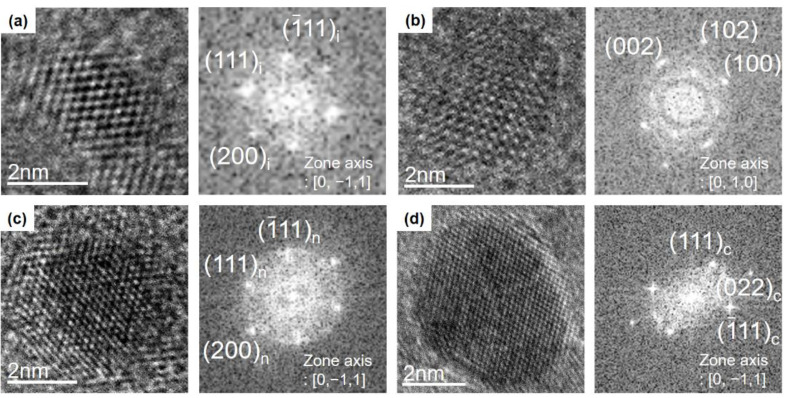
HR-TEM and FFT images of (**a**) i-carbon at 3% CH_4_-97% H_2_, (**b**) hexagonal diamond at 1% CH_4_-99% H_2_, (**c**) n-diamond at 3% CH_4_-97% H_2_, and (**d**) cubic diamond at 1% CH_4_-99% H_2_.

To characterize the phase of the nanoparticles, both d-spacings and lattice angles determined from the FFT image were compared with the reported values of carbon allotropes. The observed nanoparticles were classified into four carbon allotropes: i-carbon, hexagonal diamond, n-diamond, and cubic diamond, as shown in Figure 3, respectively. The nanoparticle in Figure 3a was captured for 10 s at 900 °C with a filament temperature at 2100 °C and gas mixture of 3% CH_4_-97% H_2_. The FFT image in Figure 3a shows the single-crystalline cubic phase diamond along the <110> zone axis. Vora et al. [18] analyzed an i-carbon film that contained an unknown cubic phase having a lattice parameter of 4.25 Å; they assigned d-spacings of 2.43 and 2.12 Å of the phase to the (111) and (200) planes, respectively. This phase is called i-carbon [19,20,21]; d-spacings of 2.42 and 2.10 Å of the nanoparticle in Figure 3a were assigned, respectively, to the (111) and (200) planes of the cubic phase with a lattice parameter 4.2 Å, which are nearly the same as the spacings reported by Vora et al. [18]. The observed i-carbon nanoparticles showed a d-spacing range of 2.36–2.54 Å. As a result, there was variation in the lattice parameter over the range of 4.1–4.4 Å. Additionally, the lattice angles of 70° and 54° shown in Figure 3a, which are between the two (111) planes and between the (111) and (200) planes, respectively, matched with those of i-carbon.

The nanoparticle in Figure 3b was captured at 300 °C with the filament temperature at 2100 °C and the gas mixture of 1% CH_4_-99% H_2_. The FFT image in Figure 3b shows the single-crystalline hexagonal diamond along the <100> zone axis. HR-TEM and FFT images show the (100), (002), and (102) lattice planes of hexagonal diamond. The lattice angles of 90° and 43° in Figure 3b, which are between the (100) and (002) planes and between the (002) and (102) planes, respectively, matched those of a hexagonal diamond structure (JCPDS No.19-0268).

The conditions used to capture the nanoparticle shown in Figure 3c were the same as those in Figure 3a, in which a gas mixture of 3% CH_4_-99% H_2_ was supplied. The FFT image in Figure 3c shows a single-crystalline n-diamond along the <110> zone axis. HR-TEM and FFT images display the (111) and (200) lattice planes of n-diamond. The lattice angles of 70° and 54° in Figure 3c, which are between the two (111) planes and between (111) and (200) planes, respectively, matched those of n-diamond (JCPDS No.43-1104).

The nanoparticle in Figure 3d was captured at 900 °C with the filament temperature at 2100 °C and the gas mixture of 1% CH_4_-99% H_2_. The FFT image in Figure 3d shows a single-crystalline cubic diamond along the <110> zone axis and the two (111) planes and the (220) plane of cubic diamond (Fd3m). HR-TEM and FFT images show d-spacings of 2.06 and 1.25 Å, which correspond to the (111) and (220) planes of cubic diamond, respectively. The lattice angles of 70° and 35° in Figure 3d, which are between the two (111) planes and between the (111) and (220) planes, respectively, match with those of cubic diamond (JCPDS No.6-0675).

### 3.2. Captured Nanoparticles under Various Processing Conditions

**Figure 4:**
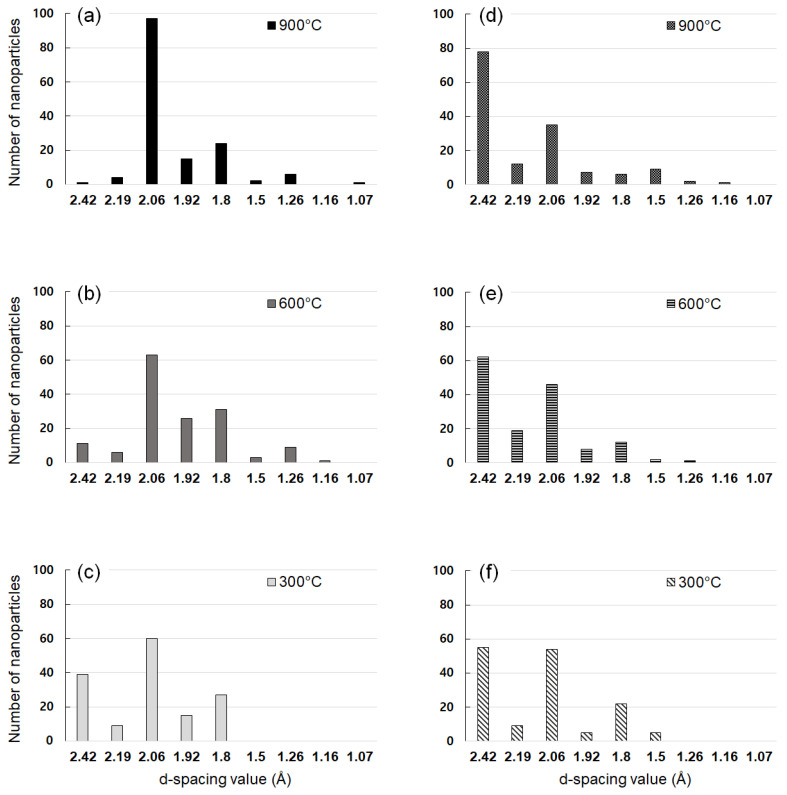
Number of nanoparticles with the given d-spacing values determined from 150 d-spacing values of ~100 nanoparticles captured at (**a**) 1% CH_4_-99% H_2_ and 900 °C, (**b**) 1% CH_4_-99% H_2_ and 600 °C, (**c**) 1% CH_4_-99% H_2_ and 300 °C, (**d**) 3% CH_4_-97% H_2_ and 900 °C, (**e**) 3% CH_4_-97% H_2_ and 600 °C, and (**f**) 3% CH_4_-97% H_2_ and 300 °C.

We tried to investigate the dependence of the crystal structure of captured nanoparticles on the capture temperature and the methane concentration. For this, we analyzed the crystal structure of the captured nanoparticles at 900 °C, 600 °C and 300 °C on the gas mixture of 1% CH_4_-99% H_2_ and 3% CH_4_-97% H_2_. Figure 4a–f show the number of nanoparticles with given d-spacing values, respectively, at 1% CH_4_-99% H_2_ and 3% CH_4_-97% H_2_ at the filament temperature of 2100 °C. The number of nanoparticles with given d-spacing values in Figure 4 was determined from 150 d-spacing values out of ~100 nanoparticles for each membrane. The number of the d-spacing values in Figure 4 would be related with the XRD intensity of the polycrystal. The number of the d-spacing values could be used to roughly estimate the phase fraction of the nanoparticles [18,19]. The d-spacing values were analyzed according to the reported values for the carbon allotrope in JCPDS. We made an approximation that the observed d-spacing would correspond to that of JCPDS if the d-spacing value coincides within ~3%.

In Figure 4a, where the gas mixture was 1% CH_4_-99% H_2_, nanoparticles captured at 900 °C show the number of 97 for 2.06 Å and the number of 1 for 2.42 Å. In addition, in Figure 4c, nanoparticles captured at 300 °C showed the number of 39 for 2.42 Å and the number of 60 for 2.06 Å. However, in Figure 4d, where the gas mixture was 3% CH_4_-97% H_2_, nanoparticles captured at 900 °C show the number of 35 for 2.06 Å and the number of 78 for 2.42 Å. On the other hand, in Figure 4f, nanoparticles captured at 300 °C showed the number of 55 for 2.42 Å and the number of 54 for 2.06 Å. The number for 2.06 Å at 1% CH_4_-99% H_2_ in Figure 4a–c is higher than that at 3% CH_4_-97% H_2_ in Figure 4d–f at all capture temperatures. 

At the gas mixture of 1% CH_4_-99% H_2_, the number of nanoparticles with 2.06 Å increases as the capture temperature increases. However, at the gas mixture of 3% CH_4_-97% H_2_, the number of nanoparticles with 2.06 Å decreases as the capture temperature increases. Figure 4a–c show that at the gas mixture of 1% CH_4_-99% H_2_, the number of cubic diamond and n-diamond increase as the capture temperature increases from 300 °C to 900 °C. On the other hand, Figure 4d–f show that at the gas mixture of 3% CH_4_-97% H_2_, the number of captured i-carbon nanoparticles increases as the capture temperature increases from 300 °C to 900 °C.

Table 1 compares the observed d-spacing values with those reported for cubic diamond, n-diamond, hexagonal diamond, and i-carbon. The relative XRD intensity (I/I_111_) is shown for each reported d-spacing in Table 1. For n-diamond, the relative XRD intensities of (111) and (200) are 100 and 100, respectively, whereas for cubic diamond, the corresponding intensities are 100 and 0, respectively. As mentioned earlier, the experimentally observed d-spacing of (200) indicates the existence of n-diamond. If nanoparticles show a high fraction of (111) but none of (200) in FFT images, then they would be cubic diamond. Although the ratio of the relative XRD intensity of (111) to (200) was 1 for the reported n-diamond in Table 1, the observed d-spacing ratio of (111) to (200) shown in Figure 4a was 5. The d-spacing ratio of the observed nanoparticles, which were captured at 900 °C with the filament temperature at 2100 °C and the gas mixture of 1% CH_4_-99% H_2_, was five-fold larger than the ratio of the relative XRD intensities. This result indicates that the nanoparticles captured at 900 °C in Figure 4a had a fraction of cubic diamond higher than that of n-diamond. 

The d-spacing of 2.42 Å belongs to i-carbon, whereas the d-spacing of 2.06 Å belongs to cubic diamond or n-diamond. Therefore, a larger number of 2.06 Å than that of 2.42 Å indicates that cubic diamond or n-diamond is a major phase with i-carbon being a minor phase. Similarly, a smaller number of 2.06 Å than that of 2.42 Å indicates that i-carbon is a major phase with cubic diamond or n-diamond being a minor phase. Figure 4 shows that the number of a 2.06 Å d-spacing for 1% CH_4_-99% H_2_ was larger than that for 3% CH_4_-97% H_2_; additionally, the number of nanoparticles for the 2.42-Å d-spacing, which was very high for 3% CH_4_-97% H_2_, is related to i-carbon. Thus, the nanoparticles for 3% CH_4_-97% H_2_ consisted mainly of i-carbon, whereas those for 1% CH_4_-99% H_2_ consisted mainly of cubic diamond or n-diamond. 

### 3.3. Size Dependence on the Crystal Structure of Nanoparticles

**Figure 5:**
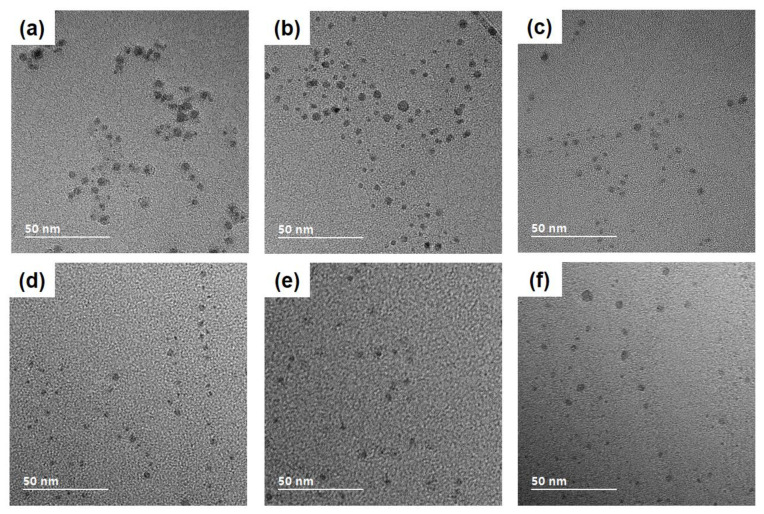
HR-TEM images of nanoparticles captured for 10 s using 1% CH_4_-99% H_2_ at (**a**) 900 °C, (**b**) 600 °C, (**c**) 300 °C and using 3% CH_4_-97% H_2_ at (**d**) 900 °C, (**e**) 600 °C, and (**f**) 300 °C.

Figure 5 shows the TEM images of nanoparticles captured under six different conditions. Figure 5a–c show nanoparticles captured, respectively, at 900 °C, 600 °C and 300 °C at the gas mixture of 1% CH_4_-99% H_2_. These images show that the size of nanoparticles decreases with decreasing capturing temperature. On the other hand, Figure 5d–f show nanoparticles captured, respectively, at 900 °C, 600 °C and 300 °C at the gas mixture of 3% CH_4_-97% H_2_. These images show that the size of nanoparticles increases with decreasing capturing temperature. To make a more quantitative analysis of the size, the size distribution of nanoparticles was measured from the TEM image. 

**Figure 6:**
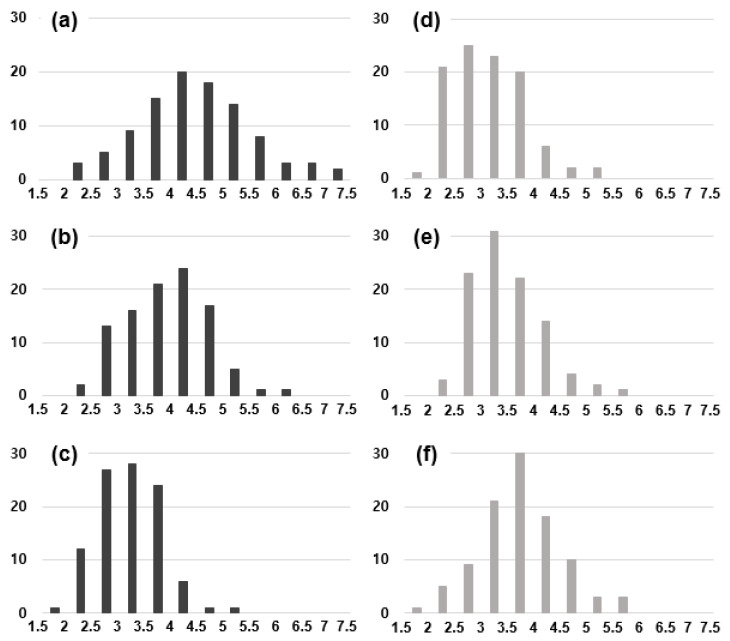
Distributions of the average size of 100 nanoparticles captured at 1% CH_4_-99% H_2_ at (**a**) 900 °C, (**b**) 600 °C and (**c**) 300 °C and captured at 3% CH_4_-97% H_2_ at (**d**) 900 °C, (**e**) 600 °C and (**f**) 300 °C.

Figure 6 shows the size distribution measured from 100 nanoparticles for each condition. At a gas mixture of 1% CH_4_-99% H_2_, when the capture temperature decreased from 900 °C to 300 °C, the average size of nanoparticles decreased from ~4.5 nm to ~3.2 nm. At 3% CH_4_-97% H_2_, however, when the capture temperature was decreased from 900 °C to 300 °C, the average size increased from ~3.1 nm to ~3.8 nm. The average size of i-carbon nanoparticles was ~3 nm regardless of the methane concentration and the capture temperature.

By comparing Figure 4 with Figure 6, it can be said that the average size of nanoparticles has a higher correlation with the number of 2.06 Å than with the capture temperature. In other words, there is a high correlation that the larger the size the larger the number of cubic diamond and n-diamond and the smaller the size the larger the number of i-carbon. This result implies the possibility that the stability of the crystal structure of nanodiamonds should depend on the size.

In relation to this possibility, we made a literature survey on the dependence of the stability of nanodiamonds on the size. As the nanodiamond size decreases, the phase stability of the diamond also changes, and the nanodiamond becomes a more stable structure than the graphite phase [22,23]. From the results, the crystal structure of the nanoparticles would be related to the size of nanoparticles. According to a previous study on the size distribution of nanoparticles produced in the gas phase of a HFCVD diamond process via a Wien filter using differential pumping, the nanoparticles contained only ~250 carbon atoms at 1.5% CH_4_-98.5% H_2_ [24]. These nanoparticles would be primary nanoparticles. The nanoparticles in Figure 5 are expected to form by coalescence of primary nanoparticles.

The question is then, why are i-carbon nanoparticles mainly formed with 3% CH_4_-97% H_2_? Moreover, why are cubic diamond and n-diamond nanoparticles more prevalent with 1% CH_4_-99% H_2_? Previously, Hwang et al. [25] suggested that the stability of diamond nanoparticles is promoted by the negative charge. To compare the amount of negative charge between the gas mixtures of 1% CH_4_-99% H_2_ and 3% CH_4_-97% H_2_, we measured the current at the location 30 mm away from the hot filament at 2100 °C using the gas mixtures of 1% CH_4_-99% H_2_ and 3% CH_4_-97% H_2_. The current measured with 1% CH_4_-99% H_2_ was −24.9 μA/cm^2^, which was almost three-fold larger than that of −8.3 μA/cm^2^ measured with 3% CH_4_-97% H_2_. These negative currents would arise from electrons emitted from the hot filament. The thermionic emission depends on the work function of the hot filament. If the filament is coated with carbon, which is shown by the phase diagram calculated by Thermo-Calc [26] to occur at 3% CH_4_-97% H_2_, the filament surface changes from tungsten carbide to graphite. Under these conditions, the work function would increase from 3.6 eV for tungsten carbide [27] to 4.6 eV for graphite [28], which could explain the three-fold discrepancy in the current readings. Relatively abundant electrons at 1% CH_4_-99% H_2_ would tend to stabilize the sp^3^ bond of nanodiamond, which would result in cubic diamond or n-diamond. On the other hand, a relative deficiency in electrons at 3% CH_4_-97% H_2_ would likely induce the sp^2^ bond of i-carbon [29,30].

### 3.4. Comparing the Captured Nanoparticles and Deposited Diamond

**Figure 7:**
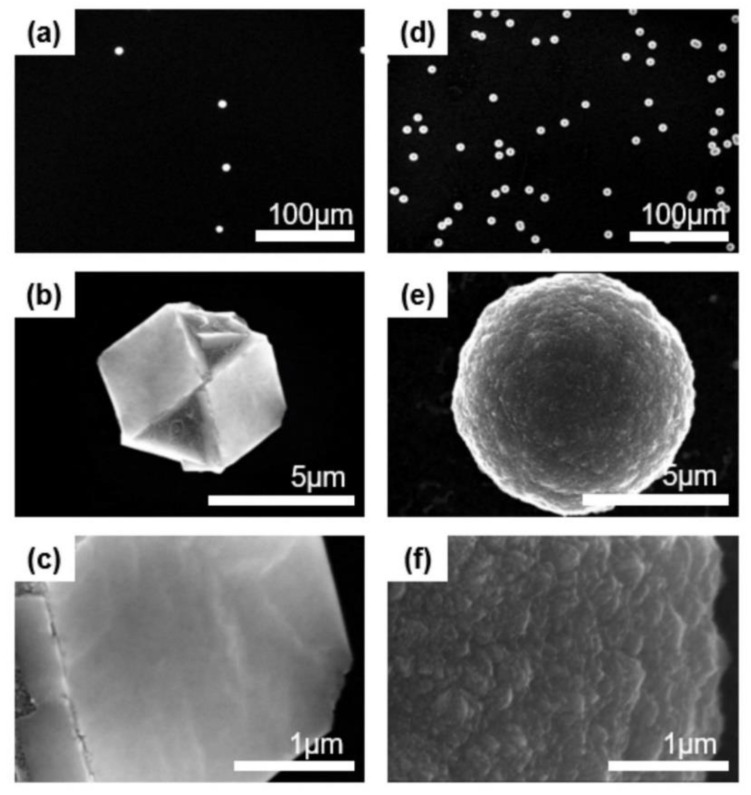
FE-SEM images of diamond deposited at (**a**) 1% CH_4_-99% H_2_ and (**d**) 3% CH_4_-97% H_2_ for 8 h on the bare silicon substrate. (**b**,**e**) are the higher magnification of (**a**,**d**), respectively, and (**c**,**f**) are the higher magnification of (**b**,**e**), respectively.

To compare deposition behavior between the gas mixtures of 1% CH_4_-99% H_2_ and 3% CH_4_-97% H_2_, a bare Si substrate without the pretreatment was used for deposition at 900 °C for 8 h under the same processing conditions as those under which the nanoparticles were captured. Figure 7 shows SEM images of diamond particles deposited on the Si substrate. Since the Si substrate was not pretreated, diamond particles instead of films were grown even after deposition for 8 h. The diamond particles deposited using the gas mixture of 1% CH_4_-99% H_2_ are shown in Figure 7a with successively higher magnification images shown in Figure 7b,c. The diamond particles deposited using the gas mixture of 3% CH_4_-97% H_2_ are shown in Figure 7d, with a higher magnification image shown in Figure 7e and an even higher one shown in Figure 7f.

The diamond particles in Figure 7a–c show well-developed (100) facets, whereas those in Figure 7d–f show ball-like or cauliflower-like structures with numerous nanosized nodules on the surfaces. The particle size in Figure 7b is smaller than that shown in Figure 7e. When comparing the crystal structures of captured nanoparticles and diamond particles deposited for 8 h, the nanoparticles captured at 1% CH_4_-99% H_2_ were grown as well-faceted diamond particles, whereas the nanoparticles at 3% CH_4_-97% H_2_ were grown as cauliflower diamond particles. These results suggest that the crystal structure of the nanoparticle affects the crystal structure of the deposited diamond. 

## 4. Conclusions

About 600 nanoparticles captured in the HFCVD diamond process at various experimental conditions were analyzed by HR-TEM and FFT images and identified as cubic diamond, n-diamond, hexagonal diamond and i-carbon. The cubic diamond and n-diamond become more stable as the size increases and i-carbon becomes more stable as the size decreases in the size range of 2~7 nm of nanoparticles generated in the gas phase of HFCVD. Diamond crystals deposited for 8 h under the condition of the capture temperature of 900 °C and 1% CH_4_-99% H_2_, where mainly cubic diamond and n-diamond nanoparticles were captured, have well-developed (100) facets, whereas diamond crystals deposited for 8 h under the condition of the capture temperature of 900 °C and 3% CH_4_-97% H_2_, where mainly i-carbon nanoparticles were captured, have ball-like or cauliflower-like structures.

## Figures and Tables

**Table 1:** Comparison of the experimentally observed d-spacing values with those reported for cubic diamond, n-diamond, hexagonal diamond, and i-carbon.

Cubic Diamond (Observed)		Cubic Diamond (JCPDS6-0675)			n-Diamond (Observed)		n-Diamond (JCPDS43-1104)			Hexagonal Diamond (Observed)		Hexagonal Diamond (JCPDS19-0268)			i-Carbon (Observed)		i-Carbon [18]	
d (Å)	*hkl*	d (Å)	*hkl*	I/I_111_	d (Å)	*hkl*	d (Å)	*hkl*	I/I_111_	d (Å)	*hkl*	d (Å)	*hkl*	I/I_111_	d (Å)	*hkl*	d (Å)	***hkl***
															2.42	111	2.46	111
										2.16	100	2.19	100	100				
2.06	111	2.06	111	100	2.06	111	2.06	111	100	2.06	002	2.06	002	100	2.10	200 *	2.12	200*
										1.93	101	1.92	101	50				
					1.78	200 *	1.78	200 *	100						1.73	211	1.74	211
										1.50	102	1.50	102	25	1.47	220	1.50	220
1.25	220	1.26	220	25	1.25	220	1.26	220	100			1.26	110	75			1.28	311
												1.17	103	50				
		1.07	311	16			1.07	311	100			1.07	112	50				

* Forbidden reflections in Fd3m.
